# Impact of diabetes on bleeding events in ST-elevation myocardial infarction patients after urgent percutaneous coronary intervention: A retrospective cohort study

**DOI:** 10.1097/MD.0000000000004470

**Published:** 2016-08-19

**Authors:** Huairong Zhang, Xiaowen Hu, Qian Wu, Bingyin Shi

**Affiliations:** a Department of Endocrinology, First Affiliated Hospital of Xi’an Jiaotong University, Xi’an Jiaotong University, Xi’an, Shaanxi, China; b Division of Endocrinology and Metabolism, McGill University Health Center, Montreal General Hospital, Montreal, PQ, Canada; c School of Public Health, Xi’an Jiaotong University Health Science Center, Xi’an, Shaanxi, China.

**Keywords:** bleeding complication, diabetes mellitus, mortality, ST-elevation myocardial infarction, urgent percutaneous coronary intervention

## Abstract

Patients with diabetes mellitus (DM) have more ischemic events and a decreased survival rate after percutaneous coronary intervention (PCI) than non-DM patients. However, it is unknown whether short-term or long-term bleeding events are associated with DM. We aimed to determine the impact of DM on mortality and bleeding events in ST-elevation myocardial infarction (STEMI) patients after urgent PCI.

This retrospective cohort study included 435 STEMI patients who had undergone urgent PCI between 2010 and 2013, comprising 97 DM patients and 338 non-DM patients. The primary outcomes were the 30-day bleeding and 30-day mortality rates. The median follow-up period was 2 years. Data regarding patient demographics, peri-PCI medication, and invasive procedures were compared between DM and non-DM patients. Multivariate logistic regression was applied to estimate the association between DM and bleeding events. Kaplan–Meier curves were calculated to elucidate the survival rate.

Compared with non-DM patients, DM patients with STEMI had a higher incidence of left ventricular ejection fraction <40% (17.6% vs 4.2%, *P* < 0.05), Killip class >II (11.3% vs 3.8%, *P* < 0.05), and smoking (44.3% vs 63.0%, *P* < 0.05). Similar peri-PCI medication and invasive procedures were administered in the 2 groups. The incidence of 30-day bleeding events was significantly higher for DM patients than non-DM patients (6.2% vs 0.9%, *P* < 0.05). A multivariate analysis showed that DM was strongly associated with 30-day bleeding events after adjusting for confounders. DM patients had significant increased mortality rates at both the 30-day and 2-year end points.

DM was an independent predictor for an increased risk of 30-day bleeding events and correlated with increased 30-day and 2-year mortality rates in STEMI patients with PCI. Our study has significant clinical implications for risk stratification before the application of urgent PCI.

## 1 Introduction

The incidence of acute ischemic heart disease-caused death has doubled during the past decade in China.^[1]^ Previous research has estimated that this trend will accelerate so as to result in 23 million individuals dying from myocardial infarction by 2030. ST-elevation myocardial infarction (STEMI) contributes to more than 80% of such events in this country. The admission rate for STEMI per 100,000 people increased from 3.5 in 2001 to 15.4 in 2011 in China,^[2]^ and nearly a quarter of these patients have diabetes mellitus (DM).^[3]^ Despite DM patients constituting such a significant proportion of patients with STEMI, clinical studies have scarcely addressed the differences in baseline profiles, management, and outcomes in DM patients, especially among Chinese DM patients.

DM is a well-known major risk factor for the development of cardiovascular disease, with it also aggravating the progression and eventually worsening the prognosis.^[4,5]^ The mortality rate among patients with acute myocardial infarction (AMI) was found to be 1.4-fold higher in those with DM than in those without DM.^[6]^ Moreover, cardiovascular disease is the leading cause of death for DM patients.^[7]^ Percutaneous coronary intervention (PCI) can significantly improve the prognosis in general STEMI patients, but DM patients do not benefit as much as non-DM patients. A few studies found that glycosylated hemoglobin^[8,9]^ and certain other baseline characteristics (e.g., being older, female,^[10,11]^ and having acute hyperglycemia^[12]^) were associated with adverse clinical outcomes in STEMI, but the underlying mechanisms remained unclear. On the other hand, PCI-related bleeding events were found to be closely associated with poor outcomes in STEMI.^[4]^ This situation has prompted several registered clinical studies^[13–15]^ to develop models for evaluating the risk factors for in-hospital major bleeding events. These models were developed and validated in the setting of acute coronary disease, including non-ST-segment elevation acute myocardial infarction (NSTEMI), unstable angina, and STEMI, and were not specifically designed for STEMI. Moreover, data on STEMI patients and bleeding events after hospitalization are still very limited.^[16]^ There is a gap in the knowledge about the potential link between DM and 30-day bleeding events or even those up to 2 years after urgent PCI in STEMI, especially among the Chinese population.

We therefore aimed to elucidate differences in the clinical baseline characteristics, management, and outcomes between DM and non-DM patients with STEMI after urgent PCI. It may be hypothesized that DM is a predictive factor for 30-day and 2-year bleeding events, and that bleeding events are associated with DM and increased mortality in Chinese STEMI patients after urgent PCI.

## 2 Methods

### 2.1 Study population

**Figure 1:**
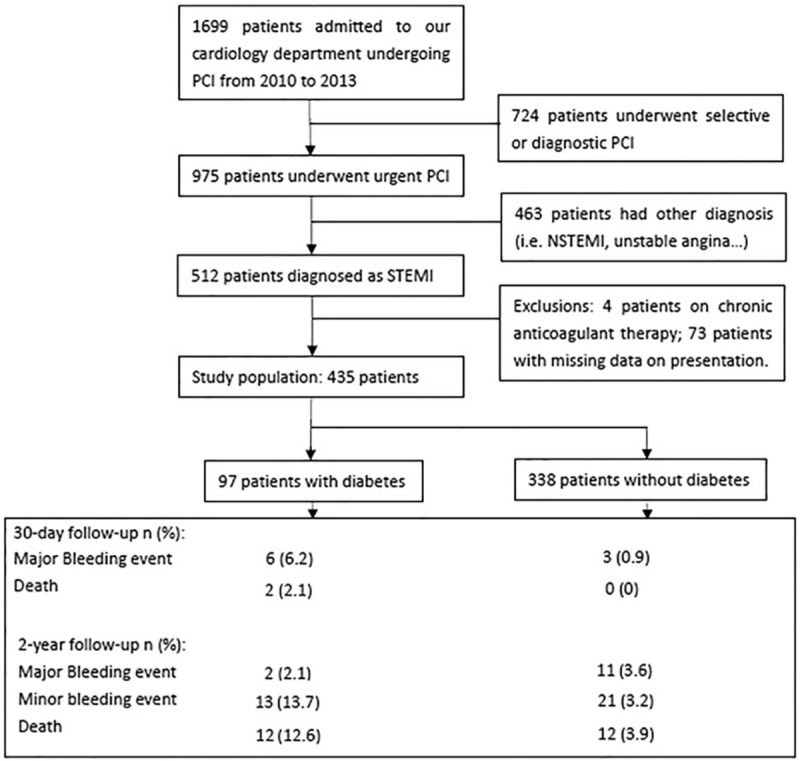
Study population selection flow diagram.

This was a retrospective cohort study. Data were obtained from the medical record database of the First Affiliated Hospital of Xi’an Jiaotong University. We included consecutive patients with STEMI who underwent urgent PCI within 12 hours after the onset of chest pain at our hospital between January 1, 2010 and December 31, 2013. The principal exclusion criteria were current use of warfarin, refusal to receive blood transfusions, gastrointestinal or genitourinary bleeding during the previous 2 months, major surgery during the previous 6 weeks, coronary stent implantation within the previous 30 days, or missing data on presentation (Fig. 1). The reports include information on patients’ demographics, clinical characteristics, peri-PCI medications, angiographic findings, invasive procedures, and clinical outcomes. The primary end points were the 30-day bleeding and 30-day mortality rates, while the secondary end points were 2-year bleeding and 2-year mortality rates. The outcomes were collected by telephone contact with the patients themselves or their relatives. During follow-up, patients were evaluated about adverse events and their compliance with antiplatelet therapy. Written consent forms were signed by all of the patients. This research protocol was approved by the Medical Ethics Committee of Xi’an Jiaotong University (approval no. KYLLSL-127-01). The study complied with the Declaration of Helsinki.

### 2.2 Definitions

STEMI was defined as chest pain lasting >30 minutes and <12 hours with documented ST-segment elevation of ≥1 mm in 2 or more contiguous leads (or reciprocal ST-segment depression of ≥1 mm in V1 or V2) or (presumed) new left bundle branch block and elevated markers of myocardial necrosis (creatine kinase MB isoenzyme or troponins).^[17]^ DM was defined as a history of hyperglycemia managed by insulin, oral hypoglycemic agents, or diet. The criteria for urgent PCI were presentation within 12 hours after the onset of chest pain suggestive of AMI and ST-segment elevation of >0.2 mV in 2 adjacent chest leads or >0.1 mV in 2 or more adjacent limb leads or >50% stenosis in an angiographic test. The patients who were finally included in the study were pretreated with a loading oral dose of 100 to 300 mg of aspirin, 600 mg of clopidogrel, and then transferred to the catheter laboratory as soon as possible. The choice of access site, antithrombotic treatment during angiography, stents, and other essential equipment was made by an experienced cardiologist in accordance with the current guidelines.^[17]^

A 30-day major bleeding event was defined as a bleeding event occurring within 72 hours after PCI or before hospital discharge and defined on the basis of the Bleeding Academic Research Consortium definition^[9]^ as the occurrence of any of the following:

1. Documented intracranial, gastrointestinal, or genitourinary bleeding.
2. Overt bleeding post-PCI plus a hemoglobin decrease of >4 g/dL. The hemoglobin decrease was calculated as the hemoglobin level examined at admission minus that tested within 72 hours after PCI during hospitalization.
3. Access-site bleeding either external or hematoma of >10 cm for femoral access, >5 cm for brachial access, or >2 cm for radial access.
4. Any bleeding requiring blood transfusion or surgical intervention for control.

A 2-year major bleeding event was defined similarly as for the 30-day period but covering any conditions that occurred during the follow-up after discharge. A 2-year minor bleeding event was defined as hemorrhage (including dental, nasal, skin, or intraocular) that did not require intervention during the follow-up.

### 2.3 Data analysis

We generated the statistics for the whole population and sorted the patients into 2 groups: DM and non-DM. Categorical variables are expressed as frequency and percentage values for Pearson *χ*^2^ tests or Fisher exact tests as appropriate. Continuous variables are expressed as mean and standard deviation values, and they were compared using Student *t* tests. The Mann–Whitney *U* test was applied to variables that did not conform to a normal distribution. Logistic regression models were applied to evaluate the prognostic effect of the DM status on bleeding events. A forward stepwise method was employed to identify covariates to be included in the logistic regression models. The adjustments included in multivariate logistic analysis were age, sex, heart rate, left ventricular ejection fraction (LVEF), and pulse pressure. Kaplan–Meier analysis was used to examine the cumulative incidence of clinical outcomes. The cutoff for statistical significance was set as *P* <0.05. All data analyses were performed using the SPSS statistical software (version 19.0; SPSS Inc, Chicago, IL).

## 3 Results

The 435 patients in the final study sample comprised 97 (22.3%) in the DM group and 338 (77.7%) in the non-DM group (Fig. 1). The follow-up period was 28.0 ± 10.2 months (mean ± standard deviation). The study population was relatively young (59.4 ± 11.2 years) and comprised predominantly men (84.6%) with a normal body mass index (BMI, 24.4 ± 3.4 kg/m^2^). Patients with DM were older (61.5 ± 11.5 vs 58.8 ± 11.1 years, *P* = 0.034) and presented with significantly lower LVEF (50.9 ± 11.3% vs 53.7 ± 10.3%, *P* = 0.028) and hematocrit (41.3 ± 6.0% vs 42.6 ± 5.5%, *P* = 0.040). The proportion of patients with Killip class >II and LVEF <40% was higher among those with DM than those without DM (11.3% vs 4.2%, *P* = 0.004, 17.6% vs 4.2, *P* < 0.001, respectively, Table 1).

**Table 1:**
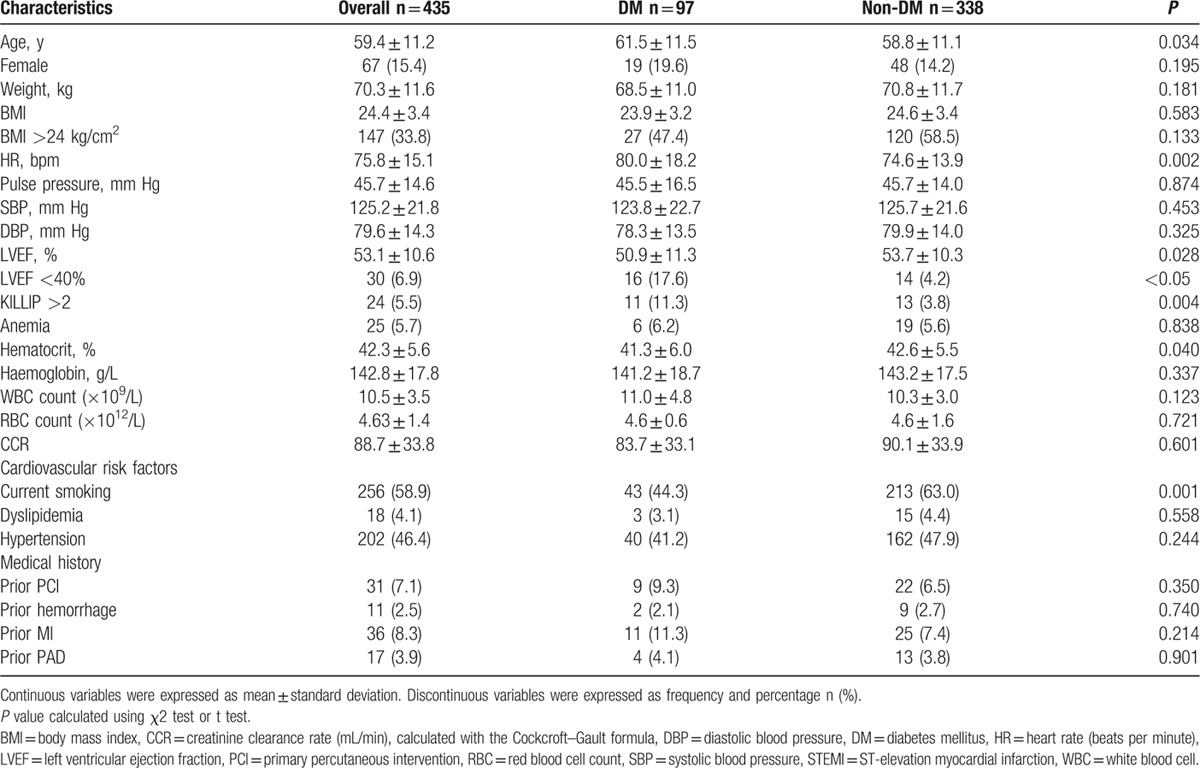
Baseline characteristics at admission in STEMI patients with or without diabetes mellitus.

Platelet GP IIb/IIIa antagonists, aspirin, clopidogrel, and antithrombin therapy were administered similarly in the 2 study groups. There were no significant differences in invasive procedures or angiographic statistics between the 2 groups (Table 2). Access-site hemorrhage was the only complication after PCI whose incidence was significantly higher in patients with DM (2.1% vs 0%, odds ratio [OR] = 1.021, 95% confidence interval [CI] = 0.992–1.051, *P* = 0.008).

**Table 2:**
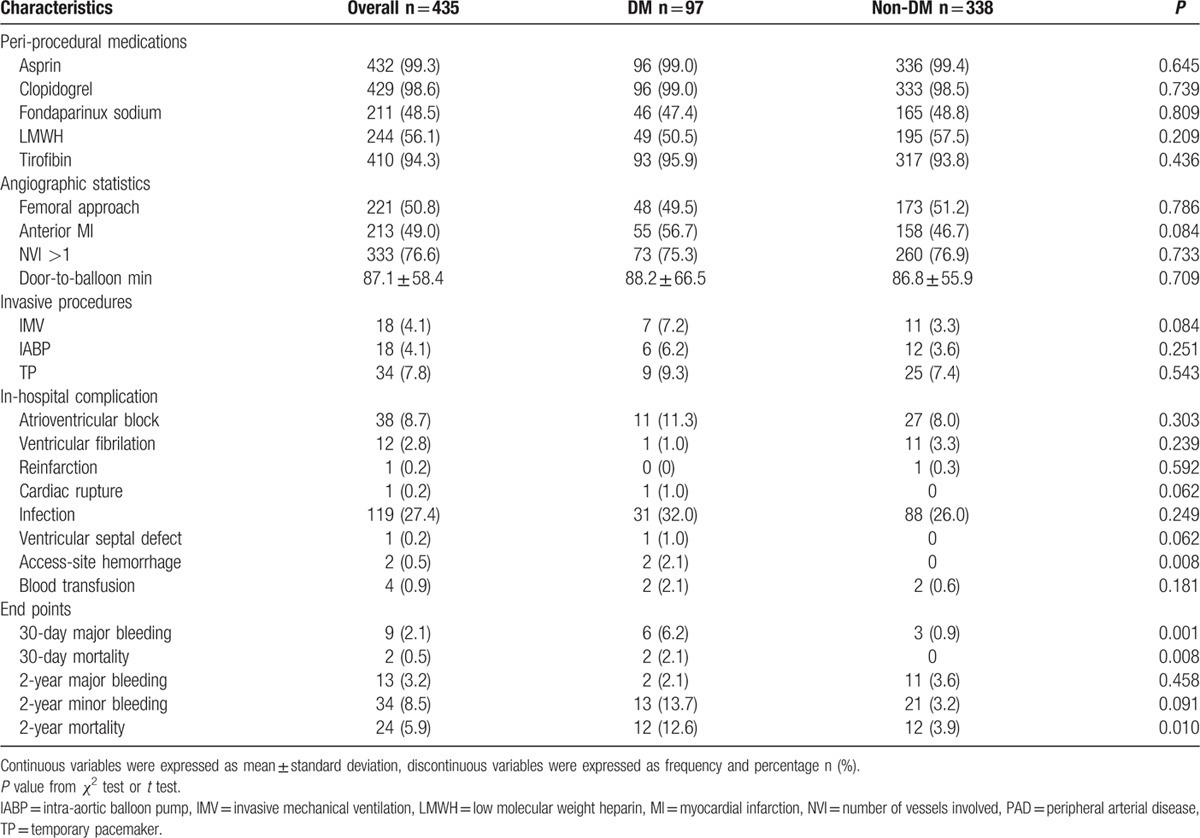
Medication within 24 hours, in-hospital procedures and clinical outcomes of the study population.

**Figure 2:**
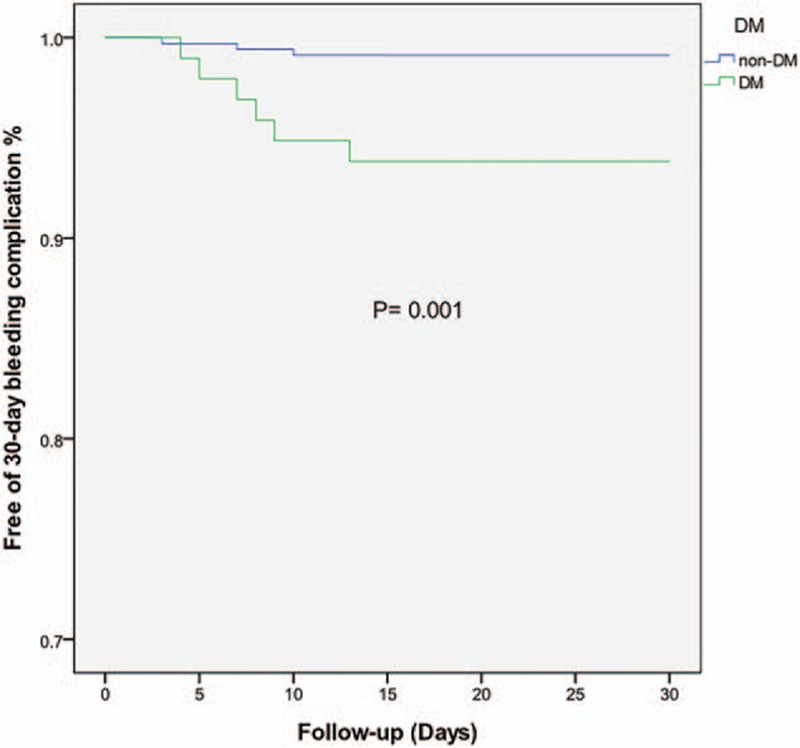
Kaplan–Meier survival curves of 30-day bleeding events in STEMI patients with or without diabetes mellitus. STEMI = ST-elevation myocardial infarction.

**Figure 3:**
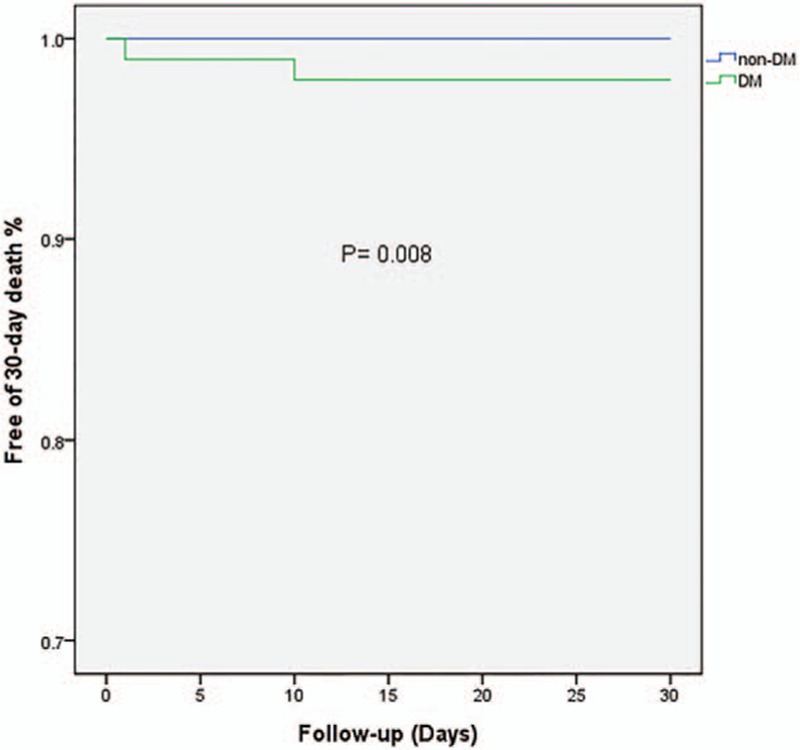
Kaplan–Meier survival curves of 30-day mortality in STEMI patients with or without diabetes mellitus. STEMI = ST-elevation myocardial infarction.

**Figure 4:**
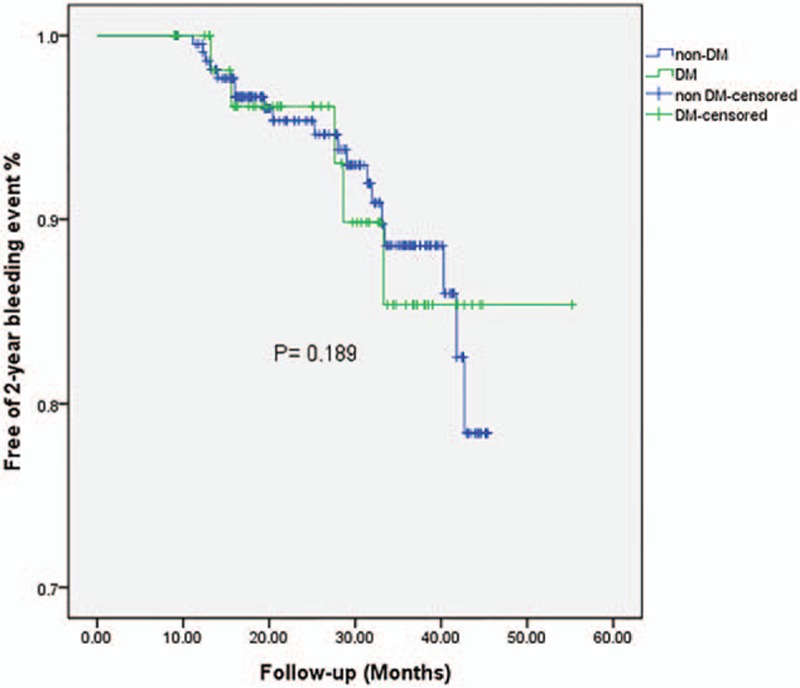
Kaplan–Meier survival curves of 2-year bleeding events in STEMI patients with or without diabetes mellitus. STEMI = ST-elevation myocardial infarction.

**Figure 5:**
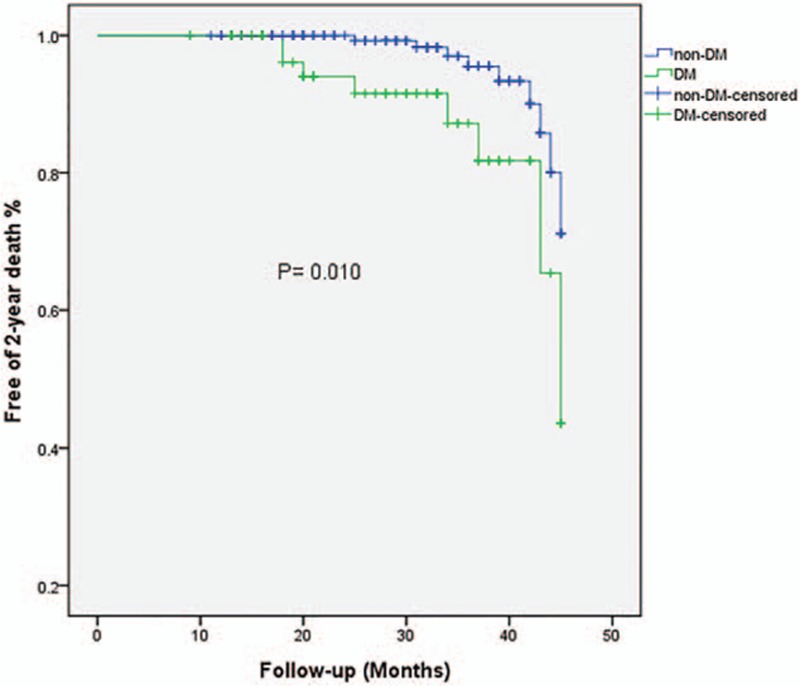
Kaplan–Meier survival curves of 2-year mortality in STEMI patients with or without diabetes mellitus. STEMI = ST-elevation myocardial infarction.

Details were obtained for 401 patients of the overall included population during the follow-up. Twenty-four patients died at the 2-year end point: 4 from myocardial reinfarction, 3 from heart failure, 3 from cancer, 4 from unknown reason, and the remainder from cardiac death. The rate of 30-day bleeding events was significantly higher in DM patients (6.2% vs 0.9%, OR = 7.36, 95% CI = 1.81–28.01, *P* = 0.005). Whether DM was an independent risk factor for 30-day bleeding events was explored by analyzing the changes in different variables relative to their baseline values as covariates (listed in Table 3). The univariate and multivariate logistic analyses of risk factors for 30-day bleeding events confirmed our hypothesis that DM was a strong predictive factor for 30-day bleeding events even after adjusting covariates (adjusted OR = 4.72, 95% CI = 1.40–15.96, *P* = 0.013). In contrast, the rates of 2-year major and minor bleeding events did not differ significant between DM and non-DM patients. Clinical outcomes (Table 2) and Kaplan–Meier curves (Figs. 2–5) implied that DM was related to significantly higher rates of 30-day mortality (2.1% vs 0.0%, *P* = 0.008) and 2-year mortality (12.6% vs 3.9%, *P* = 0.010).

**Table 3:**
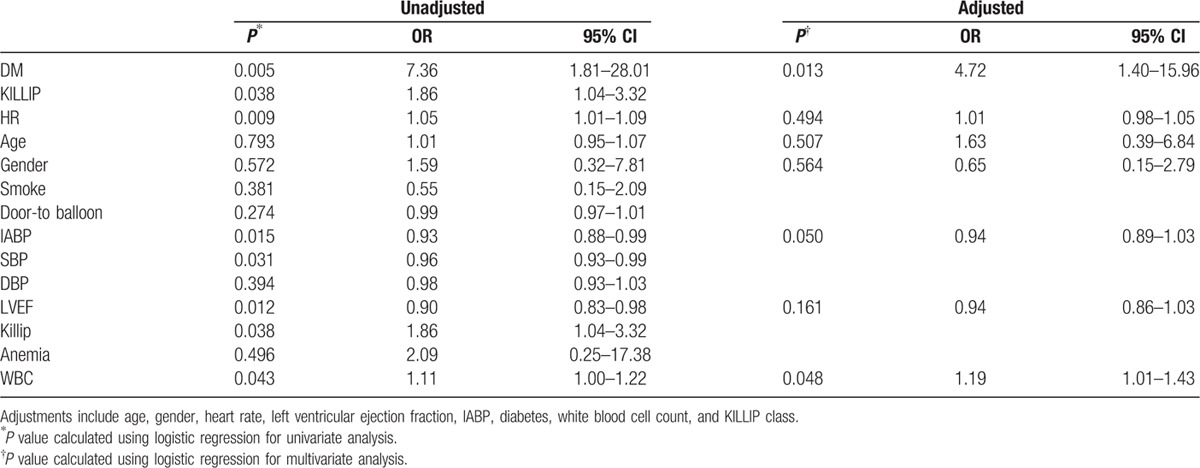
Risk factors for 30-day bleeding events.

## 4 Discussion

The main findings of our study are as follows: the hazard of a 30-day bleeding event following urgent PCI is higher in DM-related STEMI patients than in their non-DM counterparts, DM patients with STEMI undergoing urgent PCI have worse baseline profiles, more cardiovascular risk factors, but no significant difference in antithrombotic medication or in-hospital invasive procedures, and DM is significantly correlated with higher 30-day and 2-year mortality rates.

Compared with previous studies, we observed a similar prevalence of DM (22%) among STEMI patients who underwent urgent PCI (the reported rate in the patients with STEMI was 21.1% in 1 registry trial^[18]^ and 21.2% in a Chinese STEMI study population^[19]^). Consistent with previous studies,^[5,20–22]^ the DM patients in the current study and they also presented with worse baseline clinical characteristics than non-DM patients, such as being older, more likely to have previous vascular disease, more cardiovascular risk factors, higher heart rate at admission, and larger proportions in Killip class >II and with LVEF <40%. The prevalence of 30-day bleeding events was 6.2% (6/97) among DM patients, and therefore much higher than in non-DM patients. In comparison, the peri-PCI major bleeding rate in a large cohort included in a trial of acute coronary syndromes^[23]^ was 14.4% for DM patients and 10.4% for non-DM patients (*P* = 0.21). Another study, the ACUITY trial, found that DM patients—in the context of PCI—had higher rate of non-coronary-bypass-related major bleeding events (5.7% vs 4.2%, *P* <0.001).^[24]^ These disparities in the rates of major bleeding events may be due to major bleeding being defined in different ways.

DM has been validated as a risk factor for in-hospital major bleeding among patients with NSTEMI and unstable angina. However, whether DM is a risk factor for 30-day bleeding events and those up to 2 years, particularly in the Chinese population with urgent PCI for STEMI, remains unclear. As expected, the present study found DM to be an independent risk factor for 30-day bleeding events, and we attempted to identify the mechanisms underlying the increased 30-day bleeding rate. First, since DM patients usually present with previously proven predictors for bleeding, including being older and with previous bleeding events and a lower BMI, this may contribute to the interaction between 30-day bleeding and DM. Second, the use of low-molecular-weight heparin and antiplatelet agents did not differ significantly between the DM and non-DM patients, and we speculated that the short-term risk of bleeding could be higher in DM patients due to their lower body weight, especially among those who present with a significantly lower hematocrit at admission. Under this circumstance, DM patients might have higher platelet inhibition leading to reduced platelet reactivity, which increases the risk of a bleeding event.^[25]^ Third, the DM patients had been experiencing platelet hyperactivity for a long time, but they were more likely to be treated with hypoglycemic agents before AMI, which may decrease coagulability due to the reduced production of thromboxane A and plasminogen activator inhibitor 1 activity. Moreover, in addition to more frequently invasive procedures, intensive anticoagulant therapy within 12 hours before urgent PCI plus persistent treatments for hyperglycemia may enhance the burden on coagulant derangements within a short period after a clinical intervention and subsequently result in an increased bleeding rate. The above-mentioned factors may at least partially explain why the DM patients suffered more 30-day bleeding events. However, large-scale and multicenter trials involving the DM population are still needed to explore the mechanism underlying the higher bleeding rate among DM patients with STEMI after PCI. This finding indicates that DM patients should be carefully evaluated and monitored for bleeding risk. Also, the much higher bleeding rate recorded after discharge compared with the bleeding rate during 30 days after hospitalization might be due to 2-year bleeding events being covered by both major and minor bleeding definitions.

Our study has also confirmed that DM is related to higher 30-day and 2-year mortality rates. We speculate that the main reason for such higher mortality is that DM patients usually have a lower LVEF and higher Killip classification, indicating worse cardiac function and more severe cardiovascular risk factors. It was reported that Killip class >II was the most effective predictor for mortality in women with DM.^[7,26]^ We not only obtained the same association, but also extended this finding to both genders. Furthermore, the bleeding rate was significantly higher in the DM population, which is an independent risk factor for 30-day mortality among patients with coronary arterial disease.^[27]^ As mentioned above, our data indicated that both the 30-day bleeding and 30-day mortality rates were significantly higher in DM patients. It is logical and reasonable to deduce that DM elevated the 30-day mortality by increasing the probability of bleeding events. In the setting of a 2-year follow-up, DM patients do present worse mortality statistics, while the incidence of bleeding events is similar in the 2 groups. It is especially noteworthy that this parallel increase in bleeding events and mortality did not apply for the 2-year follow-up, which suggests that key risk factors involved in the disease change during its progression. We therefore assume that DM exerts time-dependent effects on bleeding events. During the short-time period around the onset of STEMI, DM was the most important risk factor for 30-day bleeding events in our study population, and it may still play a role in bleeding events by disrupting the balance between coagulation and anticoagulation during the 2-year follow-up—but it no longer acts as the key factor. Future research should include more time points when monitoring the outcome for bleeding events during the follow-up to reveal the mechanism underlying the relationship between DM and mortality.

We also found that an elevated white blood cell count at admission was related to a higher 30-day bleeding rate. This observation is identical to that of a previous study that assessed the risk factors for major bleeding after PCI.^[28]^ Finally, we found that the use of an IABP had a borderline significant predictive effect on 30-day bleeding events. A recent meta-analysis^[29]^ of the use of an IABP on AMI found that an IABP may exert beneficial effects on hemodynamic parameters, but there are no convincing large-scale data that prove this effect. Other clinical parameters previously identified as predictors for major bleeding such as advanced age and renal disease were not found in either our univariate or multivariate analysis.^[30,31]^ The smallness of the study sample and the small numbers of events may have prevented the detection of other predictors. Several other limitations of our study should also be declared. This was a single-centered study and diabetes was diagnosed at admission according to prior history but not by glucose or insulin level tests. Additionally, further study is needed about the impact of diabetes on the prognosis at different time points.

## 5 Conclusions

DM patients treated with urgent PCI for STEMI had worse baseline profiles, a higher rate for 30-day bleeding events, and impaired 30-day and 2-year survival rates. These findings indicate that DM is an independent risk factor for 30-day bleeding events. The white blood cell count before PCI and the use of an IABP were also significant factors for 30-day bleeding events. It is essential to monitor DM patients closely for bleeding events to improve their 30-day and 2-year survival rates.
